# Proceedings of the Central New York Medical Association

**Published:** 1889-01

**Authors:** Edward B. Angell

**Affiliations:** Rochester, N. Y.


### *PROCEEDINGS OF THE CENTRAL NEW YORK MEDICAL ASSOCIATION.*

Reported by EDWARD B. ANGELL, M. D., Rochester, N. Y.

The twenty-first semi-annual meeting of the Central New York Medical Association was held in the City Hall, Rochester, Tuesday, November 20, 1888.

### MORNING SESSION—10 O'CLOCK A. M.

The President, DR. WM. C. BAILEY, of Albion, called the meeting to order; the Secretary, DR. J. N. ARNOLD, of Clyde, being at his post.

DR. ELY VAN DE WARKER, of Syracuse, read a paper on "The Treatment of Stenosis of the Cervix Uteri," in which he dwelt upon the unsatisfactory results usually obtained by various methods of forcibly dilating the cervix. Ordinarily, any one of these modes gave transient relief of the symptoms, but a permanent cure seemed impossible, except when confirmed by pregnancy and parturition. This he accounted for by reason of the anatomical structure of the cervix, and the normal contractility with which the uterus is endowed. Slow dilatation might stretch the canal fully an inch without rupturing the fibers, while more rapid divulsion would do but slight damage, and, in either case, the uterus would rapidly exert its functional power and, by contracting, obliterate every evidence of the operative measure. He had seen all traces disappear in a week, and the symptomatic cure not persist to exceed two menstrual periods. The use of tents was not more satisfactory, though more dangerous, while graduated dilators were equally inefficient.

Graduated dilatation was useful when a patient declined an operation, in which instances he employed a set of graduated urethral sounds over a long period of time, maintaining a fair degree of dilatation—say 26 or 29 F.—by using one of these sizes, once a month, a few days before the expected menstruation. These failures in securing lasting results determined a very material modification of the operation. Believing that connective tissues only were ruptured by the stretching, while the muscular fibers were unaffected, and that, to secure permanent results, they, too, must be divided, he modified the operation as follows: Forcible dilatation was carried to the point where considerable resistance was encountered, the seat of greatest resistance being the os internum. Just at this stage, then, he incised the cervical canal, beginning at the os internum, and carrying it down to the os externum, employing for the purpose an Otis male urethrotome. When the instrument was expanded to the extent of 35 to 49 mm., as indicated by the dial, the blade was withdrawn until it appeared at the os externum. When the stenosis was complicated by some degree of flexion, the incision was made in the direction of the convexity of the canal, or in the angle of flexion. The large dilator was then introduced, and the stretching resumed. A comparatively slight degree of force was required to complete the dilatation to the full extent of the blades, and the operation was ended. Rupture of the os externum should be avoided, the degree of stretching being governed by the size of the cervix. The hemorrhage would be slight, and not need attention. The dilated cervix should be plugged with iodoform gauze, and the patient put to bed, with hot-water bottles at her extremities. About the third day the work should be resumed. With the vagina rendered as antiseptic as possible, the cervical canal should be gently stretched every other day with the Erlanger dilator, inserted just within the internal os. Each time the patient should be put to bed, and hot-water bottles placed at the extremities; antisepsis, warmth and rest being the factors of safety. About twenty days would be required for the treatment. Regarding results, pregnancy had followed this operation, in many instances, after years of sterility. Dysmenorrhea had been relieved for from two to four years. Yet contraction would be sure to recur, it being only a question of time, though the introduction of a moderate-sized dilator every two to four months, after the first year, would be of very material value.

Regarding diagnosis, subjective symptoms should not be convincing, unless confirmed by an exploration.

Simple "pin-hole os," when membranous, might generally be relieved by a crucial incision; but, when extending up the canal one-quarter of an inch or more, it was better to dissect the hardened margin of the os, rather than dilate it, as it was very sure to contract.

In stenosis due to flexion of the cervix upon the body of the uterus, the writer had not seen cause to change his practice of the past seventeen years, of employing the intra-uterine stem. A few simple rules required careful attention. He made sure there were no uterine adhesions, and no coincident pelvic inflammation. Toleration by the uterine cavity for a foreign body was gained by passage of the sound, at intervals of two or three days, under proper antisepsis. If dilatation was demanded before the stem could be introduced, it was better to wait till any wound that might have been made was repaired. Without further dilatation, the stem was then introduced, gentleness and patience being more requisite than force. The sound should be used to straighten out the flexion, and the stem immediately slipped in, though, in some cases, repeated introduction of the sound might be demanded before the stem would pass the angle of flexion. The stem and instruments must be thoroughly sterilized and the vagina rendered as antiseptic as possible. With these precautions, the author had not seen any evil consequences, and more satisfactory results than by other methods.

Dr. John O. Roe, of Rochester, read a paper on "Stricture of the Œsophagus, with the Report of a Case":

A lady fifty-five years old consulted him in April, 1888, with pronounced dysphagia, from which she had suffered a long time. She had already been treated by another, who practiced dilatation by bougies, but during the preceding two months the difficulty in swallowing had become so increased as to impair nutrition, and she now consulted the author. He tried dilatation for a time with gum-elastic bougies, as the stricture was accessible, it being located about two inches below the larynx. It soon became necessary to feed her through a tube, which, however, only prolonged her life about two weeks, death evidently resulting from asthenia. At the post mortem it was found that the narrowing of the œsophagus was apparently due to interstitial inflammatory thickening of its walls, and this was confirmed by the microscope. There were no evidences of cancer, and no history indicating that caustic substances had been swallowed at any time, nor that the lodgment of a foreign body had caused the inflammatory action.

Dr. Edward B. Angell, of Rochester, read a paper upon "Epidemic Diseases, with special reference to the infectious members of that class." Infectious diseases were sub-divided into three groups: the *miasmatic*, of which malarial fevers forms the type; the *contagious*, represented by measles, small-pox, and other well-known diseases; and the *miasmatic contagious*, of which typhoid fever is the most prominent representative. In miasmatic diseases, the specific excitant was propagated outside of a diseased organism, and could not transmit the disease from an infected to a sound person. In contagious disease, the poison was conveyed from one individual to another by contact, mediate or intermediate, and thus extended the specific malady. In the third group, a union of the two conditions seemed necessary for the propagation of the specific disease, a previously affected organism and an external development.

The writer then discussed the relation of the germ theory of disease to this class, calling attention to the well-known facts supporting this theory, and concluding with the statement of John Simon, "that, though it would be premature at least to say of these diseases, they certainly have, as their contagia, microphytis respectively specific to them, it seems at present not too much to say that probably such will be the case."

The various diseases at present considered as epidemic, their modes of origin and propagation, together with the social conditions favoring their spread, were taken up and discussed in turn. For the prevention of such diseases, vigorous quarantine laws, applicable to nations, cities, or individuals, had been established, and thorough disinfection practiced. Regarding the latter, many still committed the error of supposing deodorizing was disinfection. Only thorough dilution of the atmosphere during occupancy, and prolonged exposure to stifling, noxious gases after vacancy, could satisfactorily purify a contaminated room. All articles of clothing, all curtain hangings, and floor coverings—in short, everything that could furnish a lurking-place for infectious material—should be exposed for some time to a moist heat of over 212°, while, in the most contagious diseases, destruction of the fomites by burning alone would insure utmost safety. In all the miasmatic-contagious diseases, all discharges of the patient should be thoroughly disinfected and burned, or buried in a pit with an abundant mixture of the sulphate of iron, and never, under any circumstances, cast into the house-drain or sewer.

Dr. E. R. Armstrong, of Holly, reported an epidemic of diphtheria which prevailed in and about that village during the first half of this year.

It broke out at Hulberton, a hamlet near Holly, in November of last year. There were eight consecutive cases—two of a severe type—all recovering. Two months afterward, six members of one family in the near neighborhood were stricken down, two of whom died. Last April, the disease appeared in the village of Holly, the first case being that of a pupil in the public school, and several days afterward many more of the pupils became ill simultaneously. The disease directly became epidemic, prevailing during the months of May, June, and July. Altogether, there were eighty cases of the specific disease, and a similar number of cases of pharyngitis, in which the specific diphtheritic symptoms were lacking. During the epidemic there were nine deaths. In six cases, laryngeal symptoms were noted, while eight or ten were septic in character. Tracheotomy was resorted to in one desperate case, with a successful issue, though recovery was followed by partial paralysis. One death was from exhaustion, the remaining deaths being due to septicemia.

Throughout the whole course of the epidemic there was abundant proof of the communicability of the disease. In a few cases it might have arisen *de novo*. To prevent its spreading, the authorities made no proper efforts till the epidemic had nearly ceased. In his opinion, had satisfactory precautions been employed, the epidemic might have been materially shortened.

The plan of treatment usually adopted had involved isolation, the removal of all movable articles of furniture, thorough ventilation, free employment of vapors of carbolic acid and lime water, while the internal medication had usually consisted of a combination of the tincture of iron and potassium chlorate, with an initial mercurial purge. A few of the last cases had been treated by the bi-chloride of mercury method, with no better result. Special attention had been given to prevent its spread to other members of the family, and positive treatment had been instituted at the very onset of the malady —in the writer's opinion, a very important matter.

Dr. Creveling referred to cases where direct contagion was not the exciting cause. He questioned its easy communicability. He had also seen fatal cases, in which sloughing had occurred, when no diphtheritic deposit whatever had been noticed throughout the course of the disease.

On the conclusion of Dr. Armstrong's paper, the meeting adjourned for dinner, during which intermission Dr. Bailey entertained the officers, former presidents of the Association, and a few invited guests, with an elaborate banquet at the Powers Hotel.

### AFTERNOON SESSION—2.30 P. M.

The address of welcome was delivered by Mayor Parsons, in which he referred in fitting terms to the organization and work of the Society, and congratulated it upon having attained its twenty-first anniversary. Then followed

### THE ADDRESS OF DR. E. M. MOORE, SR.

In accordance with the wish of the President, Dr. Bailey, to recognize in some way the life-work of Dr. Moore, it had been agreed to devote the afternoon to the discussion of his original contributions to the surgical treatment of the upper extremities. While Dr. Moore had contributed much to the surgery of other parts of the body, his attention had been directed rather more to the upper extremity, and its consideration was peculiarly appropriate. This resulted in his giving a succinct lecture upon matters which he was in the habit of elaborating at far greater length in his former lectures. But even with all abbreviation possible, he spoke over two hours.

Any rèsumè of this discussion would naturally be unsatisfactory, and it affords the Journal much pleasure to announce that Dr. Moore has consented to reproduce it in a series of clinical lectures upon his own contributions to the surgery of the upper extremity. The first of this series will appear in the next number of the Journal, and will be devoted to fracture and dislocation of the clavicle.

Dr. W. S. Ely, of Rochester, read a paper on "Typhoid Fever," in which attention was mainly directed to its diagnosis, character, and medicinal treatment. He referred to the difficulty of its control by preventive measures, and the influences which favored the development and spread of the specific germ now regarded as its essential cause.

Diagnosis was often perplexing, and occasionally had only been settled by a post mortem examination. Text-books gave what migh be called composite pictures of the disease, but did not describe the particular case. General likenesses were furnished, from which we must expect wide variations. In this latitude, the fever might last five, six, or even eight weeks. The diagnosis would be aided by bearing in mind that the disease must be considered a species of which there were many varieties, embracing wide fluctuations of temperature, excess of nervous symptoms, or unusual tendencies to hemorrhages, or susceptibility to relapses, or troublesome gastric and abdominal symptoms, or disproportionate pulmonary symptoms, or troublesome sequelæ, as neuritis, atrophies, paralyses, etc., or even combinations of these variations. The more common error consisted in considering the disease a malarial disorder, or in confounding it with acute pulmonary tuberculosis, or with tubercular meningitis of children. Long experience was often required to reach a correct judgment. Several visits, with perhaps frequent daily observations of pulse, temperature, and respiration, with the most careful clinical record, might be demanded in clearing up doubtful cases. In all cases of continued fever, where visceral lesions, adequate to account for the disturbance, could be excluded, the suspicion of the existence of typhoid fever should be entertained. The lineal temperature curve of the text-books was seldom seen. Variations were to be expected, and if not found, it was because our observation had not been sufficiently close.

Again, in the opinion of those longest in practice, typhoid fever had undergone marked changes in its manifestations, and in this country it had now become an asthenic disease, though formerly often sthenic in character.

Regarding treatment, we must remember we have to deal with a disturbance which must last weeks, in which incendiary medication might be dangerous, while careful measures were necessary to conserve the patient's strength. Curing the disease must mean caring for it. Greater dependence was to be placed on nursing, air, water, diet, and quiet.

He valued milk as the main food in some form or combination, as it was better borne than any other one article. The whites of from three to twenty eggs a day could be incorporated with it. If the bowels were confined, animal broths might be substituted, on account of their laxative action in disorders where the tendency was to diarrhea.

Digitalis was useful as a heart tonic, while in very asthenic cases strychnine might be given in small doses with a favorable effect.

Regarding stimulants, he advised caution, and, when demanded, would begin with the milder forms, to be gradually displaced by whisky and brandy, if required.

He advocated very little medicine regularly, suggesting one-drop doses of carbolic acid and tincture of iodine thrice daily. Quinine and salicin would often act well in small tonic doses. Diarrhea should be controlled, but not entirely arrested. For intestinal hemorrhage he employed ice upon the abdomen, and ergot and opium internally. Surgical interference, in case of internal perforation, had shown discouraging results, but should hardly be abandoned as yet for that reason.

Temperature beyond 103° over a long period, in his opinion, was exhausting, and rapidly destructive of nervous tissue. Under these circumstances, he advised tepid sponging, or moderate doses of the antipyretics. In some cases alcohol would lower temperature, though its use was experimental.

Competent nursing was of highest importance, and the intelligent and accurate clinical record a good nurse could furnish would be of very material aid. He had found it good policy, contrary to the views entertained by many physicians, to give small quantities of food every hour, or even more frequently, if need be. Unusual frequency of administering nourishment might be rendered necessary by gastric irritability, and should then be resorted to.

Convalescence was often a most trying period, and demanded greatest circumspection, lest improper diet or exercise result disastrously.

Dr. B. L. Hovey, of Rochester, presented a paper on Colles Fracture, in which he reported a series of some thirty-six cases under his care during the past fifteen years. He was convinced of the truth of Dr. Moore's teaching regarding the nature of the injury, the mode of reducing the fracture, and the appropriate dressing.

Of his thirty-six cases, two were double. In eleven cases there was associated with the fracture of the radius, dislocation of the ulna. In all but two, perfect recovery resulted. One of the faulty recoveries was due to the unruliness of the patient—a boy; the other occurred in one of the instances of double fracture.

He then referred to the various dressings employed, and described the application of Dr. Moore's compress, strip of adhesive plaster, and sling, by which all of these cases had been treated. In addition, he made use of pasteboard splints on the dorsal and palmar sides of the arm and hand. The splints extended from the middle of the forearm to the knuckles. They were cut narrow, and so shaped that the hand was deflected toward the ulnar side. He secured them loosely by a few turns of a bandage. His reason for thus employing splints was two-fold; to give better satisfaction to the patient, and to prevent the injured hand from being carried in the other, a matter which seriously jeopardized the result. With this dressing properly applied, after the ulna had been replaced, there could be no shortening of the forearm. Where there was an unreduced dislocation of the ulna, the use of the pistol-shaped splint, so commonly employed, was most illogical. For, in this case, with the hand turned toward the ulnar side, the head of the bone was carried farther away from its articulating surface, while the neighboring ligaments were put upon the stretch, causing severe pain and inflammation, and often resulting in partial anchylosis of the wrist and finger-joints, with atrophy of the tissues. As might be seen, in thirty per cent. of his cases, the fracture was attended by dislocation of the ulna, while, he believed, Dr. Moore claimed it occurred in about fifty per cent., though not in all, as some had supposed. The diagnosis of the complication was all-important. Failure to make proper reduction could be detected by undue shortening of the forearm, by undue separation of the ulna from the radius, and unnatural prominence of the head of the ulna.

[This was one of the most successful meetings the Association ever held. Over 200 members were present, and it served as a fitting observance of the twenty-first anniversary of this flourishing organization.—E. B. A.]

